# (5*SR*,10*SR*,15*SR*)-Trimethyl 5*H*,10*H*,15*H*-diindeno­[1,2-*a*:1′,2′-*c*]fluorene-5,10,15-tricarboxyl­ate 0.167-hydrate

**DOI:** 10.1107/S1600536810049056

**Published:** 2010-12-04

**Authors:** Melissa C. Menard, Frank R. Fronczek, Steven F. Watkins, Raj K. Dhar

**Affiliations:** a Department of Chemistry, Louisiana State University, Baton Rouge, LA 70803-1804, USA

## Abstract

The title compound, C_33_H_24_O_6_·0.17H_2_O, which is commonly known as (*SR*,*SR*,*SR*)-trimethyl 1,10,19-truxentricarboxyl­ate, crystallizes as a hydrate with the water mol­ecule encapsulated between three ester groups by O—H⋯O hydrogen bonding to two of them. The water mol­ecule site is not fully occupied in the crystal studied, with a refined site occupancy of 0.167 (5). The 27-atom ring system is approximately planar, with a maximum deviation of 0.148 (1) Å, and the three ester substituents are all on the same side of this plane.

## Related literature

For general background to bucky balls and bucky bowls, see: Akada *et al.* (2006 ▶); Amick & Scott (2007 ▶); Berezkin (2006 ▶); Billups & Ciufolini (1993 ▶); Emsley (1980 ▶); Kroto *et al.* (1985 ▶); Narozhnyi *et al.* (2003 ▶); Mehta & Sarma (2002 ▶); Rao (1998 ▶); Takeda *et al.* (2006 ▶). For related structures, see: De Frutos *et al.* (1999 ▶, 2002 ▶).
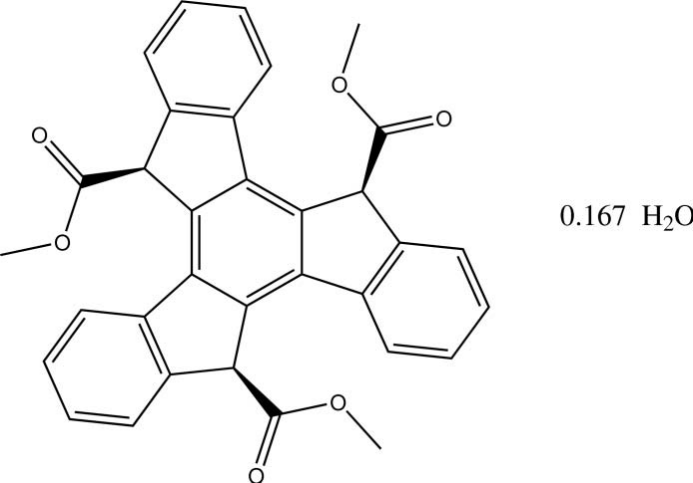


## Experimental

### 

#### Crystal data

C_33_H_24_O_6_·0.17H_2_O
*M* *_r_* = 519.61
Monoclinic, 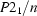

*a* = 11.8527 (3) Å
*b* = 17.3848 (5) Å
*c* = 12.6466 (3) Å
β = 96.857 (2)°
*V* = 2587.28 (12) Å^3^
*Z* = 4
Mo *K*α radiation
μ = 0.09 mm^−1^
*T* = 90 K
0.47 × 0.35 × 0.22 mm

#### Data collection

Nonius KappaCCD diffractometer with an Oxford Cryostreams Cryostream cooler
14739 measured reflections
7544 independent reflections
5513 reflections with *I* > 2s*I*)
*R*_int_ = 0.031

#### Refinement

*R*[*F* ^2^ > 2σ(*F* ^2^)] = 0.054
*wR*(*F* ^2^) = 0.144
*S* = 1.04
7544 reflections
365 parameters
H-atom parameters constrained
Δρ_max_ = 0.47 e Å^−3^
Δρ_min_ = −0.26 e Å^−3^

### 

Data collection: *COLLECT* (Nonius, 2000 ▶); cell refinement: *SCALEPACK* (Otwinowski & Minor, 1997 ▶); data reduction: *DENZO* (Otwinowski & Minor, 1997 ▶) and *SCALEPACK*; program(s) used to solve structure: *SHELXS86* (Sheldrick, 2008 ▶); program(s) used to refine structure: *SHELXL97* (Sheldrick, 2008 ▶); molecular graphics: *ORTEP-3 for Windows* (Farrugia, 1997 ▶); software used to prepare material for publication: *WinGX* (Farrugia, 1999 ▶).

## Supplementary Material

Crystal structure: contains datablocks global, I. DOI: 10.1107/S1600536810049056/bh2320sup1.cif

Structure factors: contains datablocks I. DOI: 10.1107/S1600536810049056/bh2320Isup2.hkl

Additional supplementary materials:  crystallographic information; 3D view; checkCIF report

## Figures and Tables

**Table 1:** Hydrogen-bond geometry (Å, °)

*D*—H⋯*A*	*D*—H	H⋯*A*	*D*⋯*A*	*D*—H⋯*A*
O7—H7*A*⋯O3	1.00	1.87	2.876 (9)	179
O7—H7*B*⋯O4	0.99	1.96	2.955 (9)	180

**Fig. 1.**
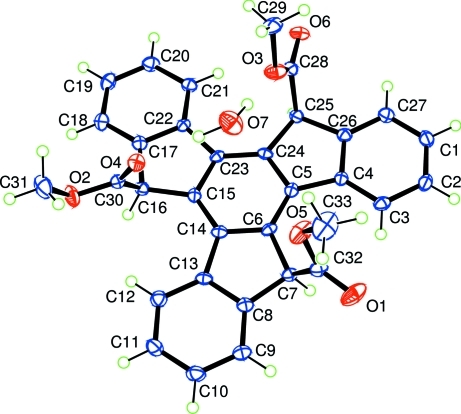
The structure of the title compound, with displacement ellipsoids drawn at the 50% probability level and H atoms with arbitrary radius.

## supplementary crystallographic information

### Comment

In 1985, Kroto, Smalley, and coworkers (Kroto *et al.*, 1985) discovered Buckminsterfullerene, also known as the bucky ball, C_60_, in the soot produced by a pulsed laser on a graphite disk. This truncated icosahedron has many interesting properties and applications that have led to the synthesis of doped and undoped C_60_ derivatives (Billups & Ciufolini, 1993; Berezkin, 2006; Takeda *et al.*, 2006; Akada *et al.*, 2006; Narozhnyi *et al.*, 2003).

Bucky bowls can be generated by opening the C_60_ cage along different symmetry pathways with the appropriate attachment of hydrogen atoms. These high symmetry bucky ball intermediates could be involved in the evolution of flat graphite to the spherical C_60_. Bucky bowls have been recognized as an important intermediate in the synthesis of C_60_. In addition to their relation to bucky ball, bucky bowls also exhibit many interesting properties including surface selective chemistry (Mehta & Sarma, 2002; Rao, 1998).

As part of an investigation of bucky bowls, C_33_H_24_O_6_. 0.17H_2_O was prepared. Synthesis by aldol condensation of indanone with NaOH yielded the trisannulated benzene (Amick & Scott, 2007). This was accomplished in the one-pot, acid catalyzed, head-to-tail cyclotrimerization synthesis method detailed in a previous report (Amick & Scott, 2007). The star ester was synthesized by treating the trisannulated benzene with *n*-butyl lithium, followed by chloromethyl formate to introduce the three ester groups. The star esters were synthesized as possible bucky bowl precursors, which when sewed together make bucky balls.

The structure, shown in Figure 1, contains three ester groups on the same side of the 27-atom ring system, such that the configurations at the asymmetric C atoms C7, C16 and C25 are all the same, *S* in the asymmetric unit of the racemic crystal. The ring system is only slightly nonplanar, with maximum deviation from its best plane 0.148 (1) Å for C16, and mean deviation 0.050 Å. The nature of the distortion from planarity is such that all three peripheral benzene rings form small dihedral angles with the central benzene ring. The ring containing C10 bends away from the central plane, forming a dihedral angle of 4.19 (7)° with the central ring, while the ring containing C19 bends in the same direction, forming a dihedral angle of 2.80 (7)° with the central ring. The third peripheral ring, containing C1, twists above and below the central ring, forming a dihedral angle of 1.98 (7)° with the central ring. Out-of-plane bending is necessary for buckybowls, and adoption of the bowl shape reduces the angle strain that would result in the planar form (Rao, 1998; Mehta & Sarma, 2002; Billups & Ciufolini, 1993). There is considerable flexibility in buckybowls, which undergo rapid inversion in solution. Corannulene, C_20_H_10_, inverts about 2×10^5^ times a second at room temperature (Rao, 1998).

One of the ester groups has the carbonyl oxygen atom O4 pointed inward, while the other two have their carbonyl oxygen atoms outward. A partially occupied [16.7 (5)%] water molecule has potential hydrogen bond contacts to the three ester groups. This water comes from the second step in the synthesis, where water is the solvent. The hydrogen bond distances are consistent with previously reported values (Emsley, 1980). Difference maps were consulted to determine the hydrogen positions. Although the disorder of the water molecule can be considered dynamic, the positional disorder of the water molecule was modeled with the two H atoms within hydrogen-bonding distance to the ester O atoms. A close contact, H29A···H7A, 2.03 Å, exists in this model, and it seems likely that when the water molecule is present, methyl group C29 probably rotates to alleviate the contact. We likely would not be able to detect the alternate positions of the methyl H atoms. The same crystal, freshly prepared, had been used in a room-temperature structure determination 15.5 years earlier. At that time, the occupancy of the water molecule refined to 45.0 (6)%, but its *U*_eq_ value was large, 0.215 (4) Å^2^, and the water H atoms could not be located.

Due to the observed stereochemistry (*syn-SR*,*SR*,*SR*)-trimethyl-1,10,19-truxentricarboxylate can be used as a bucky bowl precursor with all three ester groups on the same side of the trisannulated benzene plane. A bucky bowl could be produced by conversion of the ester groups to acid chloride groups followed by pyrolysis (Mehta & Sarma, 2002; Rao, 1998).

### Experimental

The synthesis of the truxene backbone, a triply annulated benzene ring, was accomplished by a two-step aldol condensation. Indanone, *p*-toluenesulfonic acid monohydrate, propionic acid, and *o*-dichlorobenzene were mixed in a single pot. After heating for 16 h at 378 K (105 °C), the reaction mixture was poured into methanol and slowly neutralized with NaOH. The precipitate was collected by filtration and washed. The resulting truxene, a pale yellow solid, was treated with *n*-butyl lithium to generate an anion by deprotonating the three pentlyl groups. The corresponding anion was treated with chloromethyl formate to introduce the three ester groups (Amick & Scott, 2007; De Frutos *et al.*, 1999, 2002). Crystals were grown by slow evaporation from a mixture of CH_2_Cl_2_ and methanol.

### Refinement

H atoms on C were placed in idealized positions with C—H distances 0.95 - 1.00 Å and thereafter treated as riding. A torsional parameter was refined for each methyl group. *U*_iso_ for H were assigned as 1.2 times *U*_eq_ of the attached C atom (1.5 for CH_3_ and H_2_O). H atoms for the water molecule were placed guided by difference maps, based on hydrogen bonding considerations, with O—H distance 1.0 Å, and treated as riding. The water molecule is partially occupied, and its occupancy was refined.

### Crystal data

**Table:**

C_33_H_24_O_6_·0.17H_2_O	*F*(000) = 1087
*M**_r_* = 519.61	*D*_x_ = 1.334 Mg m^−^^3^
Monoclinic, *P*2_1_/*n*	Mo *K*α radiation, λ = 0.71073 Å
Hall symbol: -P 2yn	Cell parameters from 7563 reflections
*a* = 11.8527 (3) Å	θ = 2.6–30.0°
*b* = 17.3848 (5) Å	µ = 0.09 mm^−^^1^
*c* = 12.6466 (3) Å	*T* = 90 K
β = 96.857 (2)°	Prism, yellow
*V* = 2587.28 (12) Å^3^	0.47 × 0.35 × 0.22 mm
*Z* = 4	

### Data collection

**Table:**

Nonius KappaCCD diffractometer with an Oxford Cryostreams Cryostream cooler	*R*_int_ = 0.031
ω and φ scans	θ_max_ = 30.0°, θ_min_ = 2.8°
14739 measured reflections	*h* = −16→16
7544 independent reflections	*k* = −24→24
5513 reflections with *I* > 2s˘*I*)	*l* = −17→17

### Refinement

**Table:**

Refinement on *F*^2^	0 restraints
Least-squares matrix: full	0 constraints
*R*[*F*^2^ > 2σ(*F*^2^)] = 0.054	H-atom parameters constrained
*wR*(*F*^2^) = 0.144	*w* = 1/[σ^2^(*F*_o_^2^) + (0.0635*P*)^2^ + 1.439*P*] where *P* = (*F*_o_^2^ + 2*F*_c_^2^)/3
*S* = 1.04	(Δ/σ)_max_ < 0.001
7544 reflections	Δρ_max_ = 0.47 e Å^−^^3^
365 parameters	Δρ_min_ = −0.26 e Å^−^^3^

### Fractional atomic coordinates and isotropic or equivalent isotropic displacement parameters (Å^2^)

**Table:**

	*x*	*y*	*z*	*U*_iso_*/*U*_eq_	Occ. (<1)
C1	0.79369 (14)	0.28910 (9)	0.31351 (13)	0.0230 (3)	
H1	0.822	0.2455	0.2798	0.028*	
C2	0.80979 (14)	0.29464 (9)	0.42416 (13)	0.0225 (3)	
H2	0.8484	0.2547	0.465	0.027*	
C3	0.76940 (13)	0.35885 (9)	0.47593 (12)	0.0193 (3)	
H3	0.7802	0.3629	0.5514	0.023*	
C4	0.71288 (12)	0.41658 (8)	0.41344 (12)	0.0156 (3)	
C5	0.66194 (12)	0.48950 (8)	0.44248 (11)	0.0155 (3)	
C6	0.65626 (12)	0.52438 (8)	0.54044 (11)	0.0152 (3)	
C7	0.70284 (12)	0.49746 (9)	0.65152 (11)	0.0166 (3)	
H7	0.6671	0.4473	0.6675	0.02*	
C8	0.66286 (12)	0.56068 (9)	0.72253 (12)	0.0172 (3)	
C9	0.67516 (13)	0.56468 (10)	0.83230 (12)	0.0215 (3)	
H9	0.7141	0.5255	0.8744	0.026*	
C10	0.62905 (15)	0.62756 (10)	0.87945 (13)	0.0248 (3)	
H10	0.6358	0.6308	0.9549	0.03*	
C11	0.57345 (15)	0.68576 (10)	0.81902 (13)	0.0251 (3)	
H11	0.5437	0.7285	0.8534	0.03*	
C12	0.56078 (14)	0.68193 (9)	0.70711 (13)	0.0217 (3)	
H12	0.5229	0.7216	0.6652	0.026*	
C13	0.60539 (12)	0.61825 (9)	0.65917 (11)	0.0160 (3)	
C14	0.60109 (12)	0.59552 (8)	0.54651 (11)	0.0153 (3)	
C15	0.55157 (12)	0.63095 (8)	0.45422 (11)	0.0150 (3)	
C16	0.49278 (12)	0.70854 (8)	0.43846 (11)	0.0166 (3)	
H16	0.4267	0.7127	0.4804	0.02*	
C17	0.45486 (12)	0.70999 (9)	0.31791 (12)	0.0174 (3)	
C18	0.39336 (14)	0.76566 (9)	0.25886 (13)	0.0225 (3)	
H18	0.3672	0.8102	0.2919	0.027*	
C19	0.37067 (14)	0.75483 (10)	0.14951 (13)	0.0236 (3)	
H19	0.3284	0.7927	0.1075	0.028*	
C20	0.40849 (13)	0.68979 (10)	0.10025 (12)	0.0224 (3)	
H20	0.3917	0.6837	0.0254	0.027*	
C21	0.47157 (13)	0.63294 (9)	0.16088 (12)	0.0191 (3)	
H21	0.4974	0.5882	0.128	0.023*	
C22	0.49511 (12)	0.64422 (8)	0.27088 (11)	0.0144 (3)	
C23	0.55610 (11)	0.59590 (8)	0.35456 (11)	0.0141 (3)	
C24	0.61163 (12)	0.52597 (8)	0.34860 (11)	0.0146 (3)	
C25	0.63200 (12)	0.47831 (9)	0.25254 (11)	0.0171 (3)	
H25	0.5577	0.461	0.2142	0.021*	
C26	0.69733 (12)	0.40907 (9)	0.30261 (12)	0.0174 (3)	
C27	0.73685 (13)	0.34636 (9)	0.25173 (13)	0.0213 (3)	
H27	0.7256	0.3423	0.1763	0.026*	
C28	0.70202 (13)	0.51795 (9)	0.17463 (12)	0.0205 (3)	
C29	0.85240 (15)	0.60433 (11)	0.15745 (15)	0.0320 (4)	
H29A	0.9046	0.6385	0.2012	0.048*	
H29B	0.8088	0.6342	0.1009	0.048*	
H29C	0.896	0.5644	0.1256	0.048*	
C30	0.57751 (13)	0.77331 (9)	0.46472 (11)	0.0177 (3)	
C31	0.60352 (18)	0.90226 (10)	0.51643 (17)	0.0366 (5)	
H31G	0.5615	0.9461	0.5408	0.055*	
H31H	0.6338	0.9159	0.4501	0.055*	
H31I	0.6664	0.889	0.5709	0.055*	
C32	0.83138 (13)	0.49074 (10)	0.67208 (12)	0.0208 (3)	
C33	1.00581 (15)	0.54451 (13)	0.64120 (19)	0.0433 (5)	
H33D	1.0363	0.5858	0.6002	0.065*	
H33E	1.0372	0.4951	0.6216	0.065*	
H33F	1.0269	0.5538	0.7174	0.065*	
O1	0.88051 (11)	0.44471 (9)	0.73200 (11)	0.0396 (4)	
O2	0.52757 (11)	0.83658 (7)	0.49782 (11)	0.0289 (3)	
O3	0.77526 (10)	0.56870 (8)	0.22343 (9)	0.0277 (3)	
O4	0.67626 (9)	0.76970 (6)	0.45266 (9)	0.0206 (2)	
O5	0.88321 (10)	0.54280 (8)	0.61814 (11)	0.0322 (3)	
O6	0.69483 (12)	0.50293 (8)	0.08116 (9)	0.0307 (3)	
O7	0.8591 (7)	0.6664 (5)	0.3989 (7)	0.039 (3)	0.167 (5)
H7A	0.8306	0.6321	0.3376	0.047*	0.167 (5)
H7B	0.798	0.7012	0.4172	0.047*	0.167 (5)

### Atomic displacement parameters (Å^2^)

**Table:**

	*U*^11^	*U*^22^	*U*^33^	*U*^12^	*U*^13^	*U*^23^
C1	0.0248 (8)	0.0176 (7)	0.0281 (8)	0.0014 (6)	0.0099 (6)	−0.0011 (6)
C2	0.0204 (7)	0.0191 (7)	0.0286 (8)	0.0035 (6)	0.0051 (6)	0.0074 (6)
C3	0.0170 (7)	0.0221 (7)	0.0192 (7)	−0.0025 (6)	0.0040 (5)	0.0041 (6)
C4	0.0127 (6)	0.0146 (6)	0.0200 (7)	−0.0009 (5)	0.0042 (5)	0.0022 (5)
C5	0.0114 (6)	0.0147 (6)	0.0206 (7)	−0.0011 (5)	0.0036 (5)	0.0025 (5)
C6	0.0118 (6)	0.0183 (7)	0.0152 (6)	−0.0028 (5)	0.0011 (5)	0.0049 (5)
C7	0.0157 (7)	0.0190 (7)	0.0153 (6)	−0.0014 (5)	0.0024 (5)	0.0024 (5)
C8	0.0142 (6)	0.0196 (7)	0.0180 (7)	−0.0028 (5)	0.0032 (5)	0.0011 (5)
C9	0.0215 (7)	0.0252 (8)	0.0171 (7)	−0.0017 (6)	0.0000 (5)	0.0017 (6)
C10	0.0304 (9)	0.0278 (8)	0.0159 (7)	−0.0024 (7)	0.0009 (6)	−0.0023 (6)
C11	0.0306 (9)	0.0234 (8)	0.0221 (8)	0.0005 (7)	0.0057 (6)	−0.0053 (6)
C12	0.0228 (8)	0.0202 (7)	0.0221 (7)	−0.0007 (6)	0.0025 (6)	0.0005 (6)
C13	0.0153 (7)	0.0196 (7)	0.0134 (6)	−0.0050 (5)	0.0028 (5)	−0.0006 (5)
C14	0.0137 (6)	0.0182 (7)	0.0143 (6)	−0.0049 (5)	0.0032 (5)	0.0000 (5)
C15	0.0119 (6)	0.0140 (6)	0.0197 (7)	−0.0008 (5)	0.0045 (5)	0.0010 (5)
C16	0.0159 (7)	0.0179 (7)	0.0165 (7)	0.0008 (5)	0.0037 (5)	0.0013 (5)
C17	0.0140 (6)	0.0182 (7)	0.0203 (7)	−0.0013 (5)	0.0030 (5)	−0.0001 (5)
C18	0.0209 (8)	0.0201 (7)	0.0262 (8)	0.0029 (6)	0.0010 (6)	0.0006 (6)
C19	0.0200 (8)	0.0236 (8)	0.0258 (8)	0.0044 (6)	−0.0027 (6)	0.0049 (6)
C20	0.0202 (7)	0.0287 (8)	0.0175 (7)	0.0006 (6)	−0.0009 (5)	0.0010 (6)
C21	0.0180 (7)	0.0208 (7)	0.0187 (7)	−0.0012 (6)	0.0035 (5)	−0.0006 (6)
C22	0.0118 (6)	0.0154 (6)	0.0160 (7)	−0.0010 (5)	0.0015 (5)	0.0028 (5)
C23	0.0111 (6)	0.0165 (6)	0.0149 (6)	−0.0021 (5)	0.0021 (5)	0.0040 (5)
C24	0.0130 (6)	0.0179 (7)	0.0134 (6)	−0.0037 (5)	0.0041 (5)	−0.0008 (5)
C25	0.0168 (7)	0.0195 (7)	0.0155 (7)	0.0021 (5)	0.0035 (5)	0.0008 (5)
C26	0.0152 (7)	0.0191 (7)	0.0185 (7)	−0.0010 (5)	0.0042 (5)	0.0021 (5)
C27	0.0212 (7)	0.0218 (8)	0.0214 (7)	0.0004 (6)	0.0053 (6)	0.0003 (6)
C28	0.0195 (7)	0.0227 (7)	0.0201 (7)	0.0053 (6)	0.0063 (5)	0.0032 (6)
C29	0.0229 (8)	0.0357 (10)	0.0401 (10)	0.0011 (7)	0.0145 (7)	0.0167 (8)
C30	0.0203 (7)	0.0166 (7)	0.0166 (7)	0.0001 (5)	0.0044 (5)	0.0013 (5)
C31	0.0420 (11)	0.0205 (8)	0.0513 (12)	−0.0105 (8)	0.0227 (9)	−0.0113 (8)
C32	0.0174 (7)	0.0258 (8)	0.0188 (7)	−0.0008 (6)	0.0008 (5)	0.0026 (6)
C33	0.0139 (8)	0.0498 (13)	0.0657 (14)	−0.0063 (8)	0.0023 (8)	0.0153 (11)
O1	0.0203 (6)	0.0542 (9)	0.0425 (8)	0.0028 (6)	−0.0030 (5)	0.0264 (7)
O2	0.0298 (7)	0.0161 (5)	0.0441 (7)	−0.0027 (5)	0.0183 (5)	−0.0063 (5)
O3	0.0207 (6)	0.0375 (7)	0.0260 (6)	−0.0079 (5)	0.0071 (4)	0.0051 (5)
O4	0.0171 (5)	0.0205 (5)	0.0241 (6)	−0.0015 (4)	0.0020 (4)	0.0005 (4)
O5	0.0129 (5)	0.0367 (7)	0.0464 (8)	−0.0053 (5)	0.0015 (5)	0.0181 (6)
O6	0.0430 (8)	0.0337 (7)	0.0175 (6)	0.0037 (6)	0.0124 (5)	0.0024 (5)
O7	0.029 (5)	0.048 (6)	0.040 (5)	0.002 (4)	0.003 (3)	−0.006 (4)

### Geometric parameters (Å, °)

**Table:**

C1—C27	1.389 (2)	C18—H18	0.95
C1—C2	1.393 (2)	C19—C20	1.391 (2)
C1—H1	0.95	C19—H19	0.95
C2—C3	1.407 (2)	C20—C21	1.410 (2)
C2—H2	0.95	C20—H20	0.95
C3—C4	1.398 (2)	C21—C22	1.400 (2)
C3—H3	0.95	C21—H21	0.95
C4—C26	1.398 (2)	C22—C23	1.4715 (19)
C4—C5	1.470 (2)	C23—C24	1.389 (2)
C5—C6	1.388 (2)	C24—C25	1.513 (2)
C5—C24	1.414 (2)	C25—C28	1.526 (2)
C6—C14	1.405 (2)	C25—C26	1.527 (2)
C6—C7	1.5205 (19)	C25—H25	1
C7—C32	1.519 (2)	C26—C27	1.376 (2)
C7—C8	1.530 (2)	C27—H27	0.95
C7—H7	1	C28—O6	1.2036 (19)
C8—C9	1.380 (2)	C28—O3	1.336 (2)
C8—C13	1.406 (2)	C29—O3	1.4485 (19)
C9—C10	1.388 (2)	C29—H29A	0.98
C9—H9	0.95	C29—H29B	0.98
C10—C11	1.386 (2)	C29—H29C	0.98
C10—H10	0.95	C30—O4	1.1999 (18)
C11—C12	1.407 (2)	C30—O2	1.3396 (18)
C11—H11	0.95	C31—O2	1.456 (2)
C12—C13	1.396 (2)	C31—H31G	0.98
C12—H12	0.95	C31—H31H	0.98
C13—C14	1.4736 (19)	C31—H31I	0.98
C14—C15	1.387 (2)	C32—O1	1.204 (2)
C15—C23	1.407 (2)	C32—O5	1.3274 (19)
C15—C16	1.520 (2)	C33—O5	1.448 (2)
C16—C30	1.519 (2)	C33—H33D	0.98
C16—C17	1.537 (2)	C33—H33E	0.98
C16—H16	1	C33—H33F	0.98
C17—C18	1.377 (2)	O7—H7A	1.00
C17—C22	1.399 (2)	O7—H7B	0.99
C18—C19	1.390 (2)		
			
C27—C1—C2	120.97 (15)	C18—C19—H19	119.3
C27—C1—H1	119.5	C20—C19—H19	119.3
C2—C1—H1	119.5	C19—C20—C21	120.32 (14)
C1—C2—C3	120.59 (14)	C19—C20—H20	119.8
C1—C2—H2	119.7	C21—C20—H20	119.8
C3—C2—H2	119.7	C22—C21—C20	118.18 (14)
C4—C3—C2	118.25 (14)	C22—C21—H21	120.9
C4—C3—H3	120.9	C20—C21—H21	120.9
C2—C3—H3	120.9	C17—C22—C21	119.97 (13)
C26—C4—C3	119.79 (14)	C17—C22—C23	108.91 (12)
C26—C4—C5	108.80 (12)	C21—C22—C23	131.10 (14)
C3—C4—C5	131.42 (14)	C24—C23—C15	119.79 (13)
C6—C5—C24	119.55 (13)	C24—C23—C22	130.99 (13)
C6—C5—C4	131.68 (13)	C15—C23—C22	109.21 (12)
C24—C5—C4	108.77 (12)	C23—C24—C5	120.14 (13)
C5—C6—C14	120.37 (13)	C23—C24—C25	130.15 (13)
C5—C6—C7	129.73 (13)	C5—C24—C25	109.69 (13)
C14—C6—C7	109.90 (12)	C24—C25—C28	114.97 (13)
C32—C7—C6	115.34 (12)	C24—C25—C26	102.73 (11)
C32—C7—C8	109.23 (12)	C28—C25—C26	109.61 (12)
C6—C7—C8	102.56 (12)	C24—C25—H25	109.8
C32—C7—H7	109.8	C28—C25—H25	109.8
C6—C7—H7	109.8	C26—C25—H25	109.8
C8—C7—H7	109.8	C27—C26—C4	122.10 (14)
C9—C8—C13	121.54 (14)	C27—C26—C25	127.92 (14)
C9—C8—C7	128.58 (14)	C4—C26—C25	109.98 (12)
C13—C8—C7	109.86 (12)	C26—C27—C1	118.29 (15)
C8—C9—C10	118.16 (15)	C26—C27—H27	120.9
C8—C9—H9	120.9	C1—C27—H27	120.9
C10—C9—H9	120.9	O6—C28—O3	124.25 (15)
C11—C10—C9	121.57 (15)	O6—C28—C25	123.77 (15)
C11—C10—H10	119.2	O3—C28—C25	111.92 (13)
C9—C10—H10	119.2	O3—C29—H29A	109.5
C10—C11—C12	120.44 (15)	O3—C29—H29B	109.5
C10—C11—H11	119.8	H29A—C29—H29B	109.5
C12—C11—H11	119.8	O3—C29—H29C	109.5
C13—C12—C11	118.29 (15)	H29A—C29—H29C	109.5
C13—C12—H12	120.9	H29B—C29—H29C	109.5
C11—C12—H12	120.9	O4—C30—O2	123.84 (14)
C12—C13—C8	119.98 (13)	O4—C30—C16	124.26 (14)
C12—C13—C14	131.40 (14)	O2—C30—C16	111.82 (12)
C8—C13—C14	108.58 (13)	O2—C31—H31G	109.5
C15—C14—C6	119.87 (13)	O2—C31—H31H	109.5
C15—C14—C13	131.07 (14)	H31G—C31—H31H	109.5
C6—C14—C13	109.04 (12)	O2—C31—H31I	109.5
C14—C15—C23	120.27 (13)	H31G—C31—H31I	109.5
C14—C15—C16	130.23 (13)	H31H—C31—H31I	109.5
C23—C15—C16	109.43 (12)	O1—C32—O5	123.93 (15)
C30—C16—C15	110.38 (12)	O1—C32—C7	123.98 (14)
C30—C16—C17	108.19 (12)	O5—C32—C7	112.08 (13)
C15—C16—C17	102.70 (12)	O5—C33—H33D	109.5
C30—C16—H16	111.7	O5—C33—H33E	109.5
C15—C16—H16	111.7	H33D—C33—H33E	109.5
C17—C16—H16	111.7	O5—C33—H33F	109.5
C18—C17—C22	121.96 (14)	H33D—C33—H33F	109.5
C18—C17—C16	128.51 (14)	H33E—C33—H33F	109.5
C22—C17—C16	109.52 (12)	C30—O2—C31	114.00 (13)
C17—C18—C19	118.14 (15)	C28—O3—C29	115.87 (14)
C17—C18—H18	120.9	C32—O5—C33	115.18 (14)
C19—C18—H18	120.9	H7A—O7—H7B	110.8
C18—C19—C20	121.43 (15)		
			
C27—C1—C2—C3	−0.5 (2)	C18—C17—C22—C21	−1.1 (2)
C1—C2—C3—C4	0.0 (2)	C16—C17—C22—C21	−179.71 (13)
C2—C3—C4—C26	0.4 (2)	C18—C17—C22—C23	−179.42 (14)
C2—C3—C4—C5	−179.76 (15)	C16—C17—C22—C23	1.93 (16)
C26—C4—C5—C6	−178.57 (15)	C20—C21—C22—C17	0.9 (2)
C3—C4—C5—C6	1.6 (3)	C20—C21—C22—C23	178.87 (14)
C26—C4—C5—C24	1.76 (16)	C14—C15—C23—C24	−0.8 (2)
C3—C4—C5—C24	−178.07 (15)	C16—C15—C23—C24	176.61 (12)
C24—C5—C6—C14	−0.1 (2)	C14—C15—C23—C22	178.59 (12)
C4—C5—C6—C14	−179.74 (14)	C16—C15—C23—C22	−4.01 (15)
C24—C5—C6—C7	180.00 (13)	C17—C22—C23—C24	−179.42 (14)
C4—C5—C6—C7	0.4 (3)	C21—C22—C23—C24	2.5 (3)
C5—C6—C7—C32	63.6 (2)	C17—C22—C23—C15	1.29 (16)
C14—C6—C7—C32	−116.28 (14)	C21—C22—C23—C15	−176.82 (15)
C5—C6—C7—C8	−177.77 (14)	C15—C23—C24—C5	1.0 (2)
C14—C6—C7—C8	2.33 (15)	C22—C23—C24—C5	−178.24 (14)
C32—C7—C8—C9	−61.07 (19)	C15—C23—C24—C25	−177.60 (14)
C6—C7—C8—C9	176.11 (15)	C22—C23—C24—C25	3.2 (3)
C32—C7—C8—C13	120.30 (13)	C6—C5—C24—C23	−0.5 (2)
C6—C7—C8—C13	−2.53 (15)	C4—C5—C24—C23	179.17 (12)
C13—C8—C9—C10	0.0 (2)	C6—C5—C24—C25	178.31 (12)
C7—C8—C9—C10	−178.52 (15)	C4—C5—C24—C25	−1.98 (16)
C8—C9—C10—C11	−1.0 (2)	C23—C24—C25—C28	61.1 (2)
C9—C10—C11—C12	0.9 (3)	C5—C24—C25—C28	−117.57 (14)
C10—C11—C12—C13	0.1 (2)	C23—C24—C25—C26	−179.88 (14)
C11—C12—C13—C8	−1.0 (2)	C5—C24—C25—C26	1.42 (15)
C11—C12—C13—C14	176.35 (15)	C3—C4—C26—C27	−0.5 (2)
C9—C8—C13—C12	1.0 (2)	C5—C4—C26—C27	179.63 (14)
C7—C8—C13—C12	179.78 (13)	C3—C4—C26—C25	179.02 (13)
C9—C8—C13—C14	−176.90 (14)	C5—C4—C26—C25	−0.83 (16)
C7—C8—C13—C14	1.85 (16)	C24—C25—C26—C27	179.18 (15)
C5—C6—C14—C15	0.3 (2)	C28—C25—C26—C27	−58.1 (2)
C7—C6—C14—C15	−179.79 (12)	C24—C25—C26—C4	−0.33 (15)
C5—C6—C14—C13	178.73 (13)	C28—C25—C26—C4	122.35 (13)
C7—C6—C14—C13	−1.36 (16)	C4—C26—C27—C1	0.1 (2)
C12—C13—C14—C15	0.3 (3)	C25—C26—C27—C1	−179.35 (14)
C8—C13—C14—C15	177.88 (14)	C2—C1—C27—C26	0.4 (2)
C12—C13—C14—C6	−177.92 (15)	C24—C25—C28—O6	−153.27 (15)
C8—C13—C14—C6	−0.32 (16)	C26—C25—C28—O6	91.65 (18)
C6—C14—C15—C23	0.1 (2)	C24—C25—C28—O3	29.38 (18)
C13—C14—C15—C23	−177.89 (14)	C26—C25—C28—O3	−85.70 (15)
C6—C14—C15—C16	−176.64 (14)	C15—C16—C30—O4	30.93 (19)
C13—C14—C15—C16	5.3 (3)	C17—C16—C30—O4	−80.72 (18)
C14—C15—C16—C30	66.79 (19)	C15—C16—C30—O2	−152.33 (12)
C23—C15—C16—C30	−110.26 (13)	C17—C16—C30—O2	96.02 (14)
C14—C15—C16—C17	−178.06 (14)	C6—C7—C32—O1	−148.97 (17)
C23—C15—C16—C17	4.89 (15)	C8—C7—C32—O1	96.20 (19)
C30—C16—C17—C18	−65.91 (19)	C6—C7—C32—O5	32.53 (19)
C15—C16—C17—C18	177.36 (15)	C8—C7—C32—O5	−82.31 (16)
C30—C16—C17—C22	112.63 (14)	O4—C30—O2—C31	1.1 (2)
C15—C16—C17—C22	−4.11 (15)	C16—C30—O2—C31	−175.64 (14)
C22—C17—C18—C19	0.5 (2)	O6—C28—O3—C29	−2.5 (2)
C16—C17—C18—C19	178.93 (15)	C25—C28—O3—C29	174.81 (13)
C17—C18—C19—C20	0.1 (2)	O1—C32—O5—C33	−3.9 (3)
C18—C19—C20—C21	−0.1 (3)	C7—C32—O5—C33	174.59 (16)
C19—C20—C21—C22	−0.3 (2)		

### Hydrogen-bond geometry (Å, °)

**Table:**

*D*—H···*A*	*D*—H	H···*A*	*D*···*A*	*D*—H···*A*
O7—H7A···O3	1.00	1.87	2.876 (9)	179
O7—H7B···O4	0.99	1.96	2.955 (9)	180

## References

[bb1] Akada, M., Hirai, T., Takeuchi, J., Yamamoto, T., Kumashiro, R. & Tanigaki, K. (2006). *Sci. Technol. Adv. Mater* **7**, S83–S87.

[bb2] Amick, A. W. & Scott, L. T. (2007). *J. Org. Chem.* **72**, 3412–3418. 10.1021/jo070080q 17381158

[bb3] Berezkin, V. I. (2006). *JETP Lett.* **83**, 388–393.

[bb4] Billups, W. E. & Ciufolini, M. A. (1993). *Buckminsterfullerenes*, edited by W. E. Billups & M. A. Ciufolini. New York: VCH.

[bb5] De Frutos, O., Gómez-Lor, B., Granier, T., Monge, M. A., Gutiérrez-Puebla, E. & Echavarren, A. M. (1999). *Angew. Chem. Int. Ed.* **38**, 204–207.

[bb6] De Frutos, O., Granier, T., Gómez-Lor, B., Jiménez-Barbero, J., Monge, A., Gutiérrez-Puebla, E. & Echavarren, A. (2002). *Chem. Eur. J* **8**, 2879–2890. 10.1002/1521-3765(20020703)8:13<2879::AID-CHEM2879>3.0.CO;2-4 12489216

[bb7] Emsley, J. (1980). *Chem. Soc. Rev.* **9**, 91–124.

[bb8] Farrugia, L. J. (1997). *J. Appl. Cryst.* **30**, 565.

[bb9] Farrugia, L. J. (1999). *J. Appl. Cryst.* **32**, 837–838.

[bb10] Kroto, H. W., Heath, J. R., O’Brien, S. C., Curl, R. F. & Smalley, R. E. (1985). *Nature* (London), **318**, 162–163.

[bb11] Mehta, G. & Sarma, P. V. V. S. (2002). *Tetrahedron Lett.* **43**, 6557–6560.

[bb12] Narozhnyi, V. N., Müller, K.-H., Eckert, D., Teresiak, A., Dunsch, L., Davydov, V. A., Kashevarova, L. S. & Rakhmanina, A. V. (2003). *Phys. B (Amsterdam)*, **329–333**, 1217–1218.

[bb13] Nonius (2000). *COLLECT.* Nonius BV, Delft, The Netherlands.

[bb14] Otwinowski, Z. & Minor, W. (1997). *Methods in Enzymology*, Vol. 276, *Macromolecular Crystallography*, Part A, edited by C. W. Carter Jr & R. M. Sweet, pp. 307–326. New York: Academic Press.

[bb15] Rao, H. S. P. (1998). *Resonance*, pp. 82–86.

[bb16] Sheldrick, G. M. (2008). *Acta Cryst.* A**64**, 112–122. 10.1107/S0108767307043930 18156677

[bb17] Takeda, A., Yokoyama, Y., Ito, S., Miyazaki, T., Shimotani, H., Yakigaya, K., Kakiuchi, T., Sawa, H., Takagi, H., Kitazawa, K. & Dragoe, N. (2006). *Chem. Commun.* pp. 912–914. 10.1039/b514974f 16479309

